# Clinical impact of different exosomes’ protein expression in pancreatic ductal carcinoma patients treated with standard first line palliative chemotherapy

**DOI:** 10.1371/journal.pone.0215990

**Published:** 2019-05-02

**Authors:** Riccardo Giampieri, Francesco Piva, Giulia Occhipinti, Alessandro Bittoni, Alessandra Righetti, Silvia Pagliaretta, Alberto Murrone, Francesca Bianchi, Consuelo Amantini, Matteo Giulietti, Giulia Ricci, Giovanni Principato, Giorgio Santoni, Rossana Berardi, Stefano Cascinu

**Affiliations:** 1 Oncologia Clinica c/o Università Politecnica delle Marche, Dipartimento Scienze Cliniche e Molecolari – Azienda Ospedaliera Universitaria Ospedali Riuniti di Ancona, Ancona, Italy; 2 Biologia e biochimica c/o Università Politecnica delle Marche, Dipartimento di Scienze Cliniche Specialistiche ed Odontostomatologiche – Azienda Ospedaliera Universitaria Ospedali Riuniti di Ancona, Ancona, Italy; 3 Farmacia, Università di Camerino, Camerino, Italy; 4 Dipartimento Onco-ematologia Ospedale Universitario di Modena, Università di Modena e Reggio Emilia, Modena, Italy; The Ohio State University, UNITED STATES

## Abstract

**Introduction:**

Pancreatic ductal adenocarcinoma is associated to dismal prognosis despite the use of palliative chemotherapy, partly due to the lack of knowledge of biological processes underlying disease progression. Exosomes have been identified as biomarkers sources in different cancer types. Aim of the study was to analyse the contents of circulating exosomes in patients with pancreatic cancer who received palliative chemotherapy.

**Patients and methods:**

Patients were submitted to blood sample collection before chemotherapy (T0) and after 3 months (T3). We quantified by an ELISA-based technique specific proteins of cancer-derived exosomes (CD44,CD44v6,EpCAM,CD9,CD81,Tspan8,Integrin α6,Integrin β4,CD24,CXCR4). We correlated the baseline levels of these factors and changes between T3 and T0 and survival outcomes. Survival analyses were performed by Kaplan-Meier method. Correlation was assessed by log-rank test and level of statistical significance was set at 0.05. Multivariate analysis was performed by logistic regression analysis.

**Results:**

Nineteen patients were enrolled. EpCAM T0 levels and increased EpCAM levels from T0 to T3 were those mostly associated with differences in survival. Patients having higher EpCAM had median progression free survival (PFS) of 3.18vs7.31 months (HR:2.82,95%CI:1.03–7.73,p = 0.01). Overall survival (OS) was shorter for patients having higher EpCAM (5.83vs16.45 months,HR:6.16,95%CI:1.93–19.58,p = 0.0001) and also response rates (RR) were worse (20%vs87%,p = 0.015). EpCAM increase during treatment was associated with better median PFS (2.88vs7.31 months,HR:0.24,95%CI:0.04–1.22,p = 0.003). OS was also better (8.75vs11.04 months, HR:0.77,95%CI:0.21–2.73,p = 0.66) and RR were 60%vs20% (p = 0.28). Among clinical factors that might determine changes on PFS and OS, only ECOG PS was associated to significantly worse PFS and OS (p = 0.0137and<0.001 respectively).Multivariate analysis confirmed EpCAM T0 levels and EpCAM T0/T3 changes as independent prognostic factors for PFS.

**Conclusions:**

Pancreatic cancer patients exosomes express EpCAM, whose levels change during treatment. This represents a useful prognostic factor and also suggests that future treatment modalities who target EpCAM should be tested in pancreatic cancer patients selected by exosome EpCAM expression.

## 1. Introduction

Pancreatic ductal adenocarcinoma (PDAC) represents one of the deadliest malignancies known worldwide, with less than 20% one-year survival rate [1].

Particularly in western countries, due to the increase of its incidence, an increase in mortality can be estimated in the next decades, with a number of deaths comparable with other more common cancer types such as colorectal cancer. The dismal prognosis of patients diagnosed with this disease can be traced back to the lack of early symptoms (owing to late diagnoses), the high prevalence of risk factors (such as tobacco smoking, diabetes, obesity and increased alcohol intake) and poor response to treatments. In particular, novel treatment options for patients with PDAC are lacking (with the exception of Pembrolizumab for the relatively rare number of patients who have altered mismatch repair activity in the tumour).

In the last decade advances in palliative treatment of this group of patients has mainly consisted of various chemotherapy combinations of 2 (Gemcitabine + Nab-Paclitaxel) [2], 3 (Folfirinox) [3], 4 (PEXG/PEFG) [4] different drugs over Gemcitabine monotherapy [5].

Unfortunately these treatments have yielded unsatisfactory results, with median overall survival of 9–11 months for the combination chemotherapy and even less for Gemcitabine monotherapy. The reason of these results can be partly explained by pancreatic cancer heterogeneity [6] and the poor knowledge of biological mechanisms that sustain PDAC. About the latter, despite different subtypes of variation in chromosomal structure in PDAC are known (stable, locally rearranged, scattered, unstable) [6,7] clinical implications of this classification system, for the time being, seem marginal at best. There is also a great need for reliable biomarkers to stage and assess the response to therapy of PDAC and up till now Carbohydrate Antigen 19–9 (CA19-9) is the only prognostic biomarker approved by FDA but it has limitations [8, 9].

Moreover, it should be taken into account that tumour tissue samples often lack due to the anatomical difficulty to reach the primary tumour site or the critical patients’ clinical conditions that impede to carry out invasive and repeated biopsies in order to monitor disease development.

Recently the research of biomarkers moved its attention on exosomes since their content (DNA, RNA and proteins) reflect in a dynamic way the content of cell that bud them [10–12], that is, it is not constant but reflects the status of the cell of origin.

Exosomes are vescicles secreted in the extracellular matrix and that interact with cells that absorb them, thus representing a mechanism for the paracrine-autocrine regulation in both normal and tumoural cells. Those released from cancer cells regulate microenvironment, angiogenesis and immune response [13].

By isolating exosomes from body fluids it is possible to carry out liquid biopsy, a promising and minimally invasive alternative to surgical biopsies that allows the detection of cancer biomarkers. Nowadays, molecules contained in exosomes are being evaluated to discover potential biomarkers and thus, should exosomes reliably reflect the behaviour of the disease, they might offer an interesting tool to perform reproducible and relatively rapid assessments of the current status of the tumour.

In this work we measured different populations of exosomes in plasma of PDAC patients and looked for correlation with clinical variables. For example, with exosome CD133 population we mean the amount of plasma exosomes carrying CD133 protein.

## 2. Materials and methods

### 2.1 Patients

Patients candidate to be enrolled in the analysis were those with a histologically/cytologically confirmed diagnosis of metastatic/locally advanced PDAC who were candidate to receive 1^st^ line palliative chemotherapy (either Gemcitabine+Nab-Paclitaxel, FOLFIRINOX or Gemcitabine monochemotherapy). Patients should have received a staging chest/abdomen CT scan no longer than 4 weeks since the start of chemotherapy. Patients who had previously received surgery for the primary tumour were admitted as well as patients who had received adjuvant chemotherapy if more than 6 months had passed since the last cycle of adjuvant chemotherapy and the diagnosis or disease relapse.

All patients were treated until disease progression (as per standard guidelines), with dose-reductions/chemotherapy maintenance by investigator’s choice. Patients were evaluated by RECIST 1.1 criteria as for radiological responses to treatment, scheduled at approximately 3 months (12 weeks) after the start of 1^st^ line chemotherapy and then every 3 months thereafter. For the means of this analysis we collected data concerning progression free survival (PFS) (defined as the time between the 1^st^ cycle of chemotherapy and the time of the first radiological progression or lost-at-follow-up-visit or death, whichever came first) and overall survival (OS) (defined as the time between the 1^st^ cycle of chemotherapy and patient’s death or lost-at-follow-up visit).

A written informed consent (that can be found in S1 File) was signed/subscribed by all patients that were enrolled in the study. This research was read, approved and signed by the local ethical committee of our hospital.

### 2.2 Blood sample collection

Blood samples were taken before chemotherapy treatment (T0) and after three months (T3). EDTA plasma was separated from blood cells by centrifugation at 1100g for 20 minutes at room temperature (RT). Supernatant was again centrifuged at 10000 g at 4°C for 7 minutes to eliminate microvesicles. Finally, plasma samples from each patient were stored at -80°C until use. In order to test different conditions for the optimum/best sample preservation we used Protease Inhibitor Cocktail (P8340 Sigma-Aldrich), 10μl in 1ml of plasma.

### 2.3 Antibodies

The following primary and secondary antibodies were used for exosome characterization by ELISA. Mouse monoclonal antibodies anti-human CD9 (cat. 555370), CD81 (cat. 555675), CD24 (cat. 555426), Caveolin 1 (cat. 610407) and Fibronectin (cat. 610077) were purchased from BD Biosciences, USA. Mouse monoclonal antibody anti-human TSPAN8 (cat. WH0007103M2) was purchased from Sigma-Aldrich (Milano, Italy). Mouse monoclonal antibodies anti-human CD133 (cat. MAB11331), PD-L1 (cat. MAB1561), CXCR4 (cat. MAB172), EpCAM (cat. MAB9601), Integrin α6 (cat. MAB1350), Integrin β4 (cat. MAB4060), CD44s (cat. MAB7045) and CD44v6 (cat. BBA13) was purchased from R&D System, Minneapolis, USA. Mouse monoclonal antibodies anti-human CD151 (cat. 271216) and Alix (cat. 53540) were purchased from Santa Cruz Biotechnology. Rab5b polyclonal rabbit antibody anti-human (cat. HBM-RAB5-PR1, Hansa BioMed Life Sciences Ltd and cat. 598, Santa Cruz Biotechnology) was used for plate coating. Goat anti-mouse biotin conjugated antibody (cat. A16076, Thermo Fisher Scientific) was used as secondary antibody. Streptavidin Poly-HRP (cat. 21140, Thermo Fisher Scientific) is used to amplify the signal.

### 2.4 ELISA protocol

Transparent Nunc MaxiSorp flat bottom 96-well plates (cat. 439454, Thermo Fisher Scientific) with high protein-binding capacity (600–650 ng IgG/cm^2^) were used for the ELISA.

200μl of 4μg/ml anti-Rab5b in coating buffer sodium carbonate buffer (pH 8.5) are placed in each well and incubated overnight at 4°C. Afterwards the wells were washed 3 times with PBST (0.1% Tween 20 in 1 X PBS). Blocking procedure was performed by 1% of Bovine Serum Albumine (BSA) protease and fatty acid free (cat. A7030, Sigma Aldrich) in PBS for 90 minutes at RT under shaking. After washing in PBST, 100μl plasma were incubated 2h at 37°C and then overnight at 4°C. After washing in PBST, 100μl of 1 μg/ml of primary antibodies was incubated for 3 hours at RT. After washing, 100μl of 50 ng/ml of secondary antibody was incubated for 45 minutes at RT. After washing, 100μl of 50 ng/m of Streptavidin Poly-HRP was incubated for 45 minutes at RT. Dilution buffer for all antibodies was PBS with 300 mM of NaCl, 0.05% Tween 20, 0.5% BSA. Note that the use of 300 mM of NaCl instead of 150mM resulted in an increased in specificity. The substrate solution 1-Step Ultra TMB-ELISA (cat. 34028, Thermo Fisher Scientific) was added for 20 minutes at RT under shaking and the reaction was stopped by 2N H_2_SO_4_. Optical densities were recorded at 450 nm by Multiskan FC Microplate Photometer (Thermo Fisher Scientific). Washings were done by Wellwash Versa Microplate Washer (Thermo Fisher Scientific) to assure intra-assay repeatability and incubations were done in dark conditions. To the success of the assay it is important that all buffers are freshly prepared. To adjust our protocol we used lyophilized exosomes from plasma of healthy donors (cat. HBM-PEP-100/4, HansaBioMed Life Sciences Ltd, Tallin, Estonia).

For each plate and for each kind of antibody the absorbance value of the sample (signal) was divided by the absorbance of the blank (noise) to obtain a normalized value. Blank is obtained testing PBS instead of plasma.

### 2.5 Statistical analysis

Survival analysis was conducted by Kaplan-Meier method whereas different survival outcomes among different strata were estimated by log-rank test. Multivariate analysis was conducted by cox-regression analysis.

Association among categorical variables were performed by Fisher exact test for binomial variables and Chi-square test for all other instances. For all tests level of statistical significance (alpha) was set at 0.05.

To identify different groups of patients based on their exosomes’ concentration, categorical tests (hierarchical clustering analysis and K-means analysis) were used.

All statistical analysis concerning survival and correlation between categorical variables were performed by using MedCalc Statistical Software version 14.10.2 (MedCalc Software bvba, Ostend, Belgium; http://www.medcalc.org; 2014), whereas K-means analysis and hierarchical clustering analysis were performed by using R software for Windows (3.4.2 version).

## 3. Results

Nineteen patients were eligibible for enrollment and partecipated to our study. Patients’ clinical characteristics can be found in Table 1.

**Table 1:** Patients clinical characteristics.

Patient No	Age	Gender	ECOG at treatment start	M+ at enrollment (Y/N)	TNM	Metastatic sites where	Markers at start	Chemotherapy (specify)
**1**	69	M	0	YES	pT2 pN1 cM1	Liver	CEA Negative CA19.9 Negative	Gemcitabine+Nab-Paclitaxel
**2**	44	M	1	NO	cT3 cN+ cM0	/	CEA Negative CA19.9 2062 U/ml	FOLFIRINOX
**3**	63	M	0	NO	CT4 cN+ cM0	/	CEA Negative CA19.9 414 U/ml	FOLFIRINOX
**4**	57	M	1	YES	CT2 cN1 cM1	Liver	CEA Negative CA19.9 10272 U/ml	Gemcitabine+Nab-Paclitaxel
**5**	56	M	2	YES	CT2 cN1 cM1	Liver	CEA Negative CA19.9 Negative	FOLFIRINOX
**6**	68	M	0	YES	CT2 cN1 cM1	Liver	CEA Negative CA19.9 797 U/ml	Gemcitabine+Nab-Paclitaxel
**7**	73	M	0	YES	CT3 cN1 cM1	Liver	CEA Negative CA19.9 1733 U/ml	Gemcitabine+Nab-Paclitaxel
**8**	58	M	0	YES	CT3 cN0 cM1	Lung	CEA 29 ng/ml CA19.9 45 U/ml	Gemcitabine+Nab-Paclitaxel
**9**	65	M	2	YES	Ct3 cN+ cM1	Liver, Peritoneum	CEA negative CA19.9 >100.000U/ml	Gemcitabine+Nab-Paclitaxel
**10**	75	M	1	YES	CT2 cN0 cM1	Liver	CEA 211 ng/ml CA19.9 11997 U/ml	Gemcitabine
**11**	61	M	2	YES	CT4 cN1 cM1	Liver	CEA 200 ng/ml CA19.9 47200 U/ml	Gemcitabine+Nab-Paclitaxel
**12**	59	M	0	NO	CT4 cN1 cM0	/	CEA negative CA19.9 2954 U/ml	Gemcitabine → Capecitabine+RT
**13**	65	M	0	YES	PT3 pN1 cM1	Liver	CEA negative CA19.9 99 U/ml	Gemcitabine+Nab-Paclitaxel
**14**	54	M	0	YES	CT43 cN1 cM1	Liver	CEA negative CA19.9 385 U/ml	Gemcitabine+Nab-Paclitaxel
**15**	60	M	0	YES	CT3 cN0 cM1	Liver	CEA negative CA19.9 negative	Gemcitabine+Nab-Paclitaxel
**16**	71	F	1	YES	CT4 cN0 cM1	Liver, Lung	CEA 62 ng/ml CA19.9 26050 U/ml	Gemcitabine+Nab-Paclitaxel
**17**	61	M	0	NO	CT4 cN1 cM0	/	CEA negative CA19.9 5048 U/ml	FOLFIRINOX
**18**	72	M	2	YES	PT3 pN1 cM1	Peritoneum	CEA negative CA19.9 negative	Gemcitabine
**19**	76	M	2	YES	cT3 cN1 cM1	Peritoneum, Lung	CEA negative CA19.9 2703 U/ml	Gemcitabine

In particular, in the enrolled population, 15 (79%) had metastatic disease at the time of enrolment whereas the remaining 4 (21%) patients had an unresectable locally advanced pancreatic cancer. 11 (58%) patients received 1^st^ line chemotherapy with Gemcitabine+Nab-Paclitaxel, 4 (21%) received FOLFIRINOX and the remaining 4 (21%) patients were treated with Gemcitabine monotherapy.

Most of patients had ECOG:0 status at the time of enrolment (10/19, 50%) and the primary site of metastatic involvement was the liver (10 patients had the liver as the only site of metastatic involvement). Only 3 (16%) patients had received surgery with radical intent on the primary tumour and subsequently relapsed.

While all patients who were enrolled in the analysis received first line chemotherapy, only 12 (63%) received 2^nd^ line therapy and an additional 3 (16%) patients also received 3^rd^ line treatment.

During the follow-up period ALL patients did experience disease progression whereas 15 (79%) patients have died.

### 3.1 Overall population survival analysis

Among our patients, none patient experienced complete response, 8 (42%) partial response, 3 (16%) disease stabilisation, 4 (21%) disease progression due to the occurrence of new metastatic sites (whereas the primary tumour and the pre-existing metastatic sites remained stable or even in partial response) and the remaining 4 (21%) experienced disease progression in metastatic sites of involvement that were already known at the beginning of the observation.

In the group of patients with proper metastatic involvement (15/19), 6 (42%) achieved partial response, 2 (14%) achieved stable disease, 3 (21%) progressed due to the occurrence of other metastatic sites of involvement and the remaining 4 (21%) experienced disease progression in sites of metastatic involvement that were already known at the time of enrolment.

Median overall survival was 8.74 months and median progression free survival was 3.80 months.

Looking at stratification factors that might have influenced progression free survival and overall survival:

ECOG performance status was found to be a factor associated with worse progression free survival (mPFS respectively among 0-1-2 of 6.29 vs 3.01 vs 0.56 months, p = 0.0137) and with worse overall survival (mOS respectively 12.13 vs 5.80 vs 3.47 months, p<0.0001). ECOG was not associated with disease response/stabilisation/progression (p = 0.16).

Patients that received surgery for the primary tumour were not found to be associated with different progression free survival (p = 0.69), overall survival (p = 0.74) and disease response/stabilisation/progression (p = 0.56).

Type of chemotherapy used (Gemcitabine vs Gemcitabine+Nab-Paclitaxel vs FOLFIRINOX) was not associated with a statistically significant difference in terms of mPFS (p = 0.55) albeit patients treated with Gemcitabine+Nab-Paclitaxel experienced the longest mPFS (5.73 months). No difference in terms of overall survival was found (p = 0.47), albeit patients treated with Gemcitabine+Nab-Paclitaxel had the longest median overall survival (8.03 months). There was also no association with significantly different response rates (p = 0.32).

As it could be expected, patients who received 2^nd^ line chemotherapy had greater overall survival compared with patients who just received only first line chemotherapy (11.04 vs 5,92 months, HR:0.25, 95%CI:0.06–1.17, p = 0.0027). This difference was evident also (albeit not statistically significant owing to the low number of patients), between those who received also 3^rd^ line chemotherapy compared with those who received only first line treatment (16.45 vs 6.98, HR:0.46, 95%CI:0.14–1.47, p = 0.19).

We did not find any differences in terms of median progression free survival times between patients with high values of CA19.9 vs those who did have negative values of CA19.9 (despite having metastatic disease) (p = 0.34). Overall survival was also not significantly different (p = 0.75) as well as different profile of response (p = 0.43).

### 3.2 Exosome analysis

Blood samples were collected from PDAC patients before (T0) and three months (T3) after chemotherapy treatments. Through our ELISA assay we measured levels of different exosome populations, in particular those carrying the ubiquitous CD9, CD81 and some markers already found in tumour exosomes of pancreatic cancer such as CD44v6, Tspan8, EpCAM, CD24, CXCR4, Integrins α6 and β4, CD133.

During the phase of protocol optimization, we assured that the storage of plasma at -80°C minimally affect the measures, in particular, results of stored sample were smaller than the fresh samples within 10%. The addition of protease inhibitors to plasma before storing did not restore the values. However, since the lowering of the signal was slight (<10%) we stored the samples without protease inhibitors. The absorbance values were multiplied by 1000 and successively normalized dividing each sample values with the blank value. Results are reported in the Table 2. Since CD151, Caveolin-1, PD-L1, Alix, CD133 and Fibronectin antibodies were not used for all enrolled patients, correlations of their levels and clinical features were carried out only for CD9, CD81, EpCAM, Integrin α6, Integrin β4, CD44s, CD44v6, CXCR4, Tspan8 and C24.

**Table 2:** Results of exosomes’ protein evaluation respectively for T0 (baseline) and T3 (after 3 months of treatment).

**Pts**	**CD44 T0**	**CD44v6 T0**	**CD24 T0**	**CD9 T0**	**CD81 T0**	**EpCAM T0**	**Integrin α6 T0**	**Integrin β4 T0**	**Tspan8 T0**	**CXCR4 T0**
**1**	3.6	2.1	2.3	14.5	3.4	3.2	9.6	2.4	1.7	5.2
**2**	4.9	4.5	4.6	5.5	4.7	6.3	5.7	5.3	4.2	5.5
**3**	2.5	1.6	1.8	6.2	2.8	2.5	3.9	1.8	1.4	5.1
**4**	3.4	2.0	2.1	8.3	3.5	2.9	3.3	2.3	1.9	5.3
**5**	3.3	2.5	4.3	18.6	3.0	21.4	4.4	6.5	1.9	17.5
**6**	2.6	3.6	2.6	26.5	5.1	1.2	2.5	2.8	1.8	5.9
**7**	4.0	3.6	2.8	35.5	14.7	1.0	2.1	3.0	1.7	8.7
**8**	7.6	2.7	8.3	13.9	8.9	8.3	2.8	3.0	3.0	3.8
**10**	8.0	3.1	2.9	11.2	9.8	2.1	7.0	3.4	1.7	9.1
**11**	4.0	2.2	3.5	17.9	5.8	2.8	2.3	1.8	2.2	2.5
**12**	3.3	1.5	1.5	10.3	4.4	1.2	8.7	1.9	0.9	8.1
**13**	2.6	3.5	6.3	15.9	6.6	1.4	1.5	1.7	1.8	1.5
**14**	1.1	1.3	5.3	19.7	12.4	2.0	1.5	1.0	1.0	2.2
**15**	7.8	1.3	3.1	7.3	5.8	2.2	1.3	1.1	1.3	2.6
**16**	9.2	2.0	10.2	26.3	8.4	2.6	2.6	1.5	1.2	2.9
**17**	3.9	1.9	6.1	22.5	3.8	3.3	1.8	1.2	2.1	2.7
**18**	7.5	2.9	3.4	33.2	10.1	3.4	3.7	5.7	2.2	4.4
**19**	8.2	3.5	6.2	32.9	8.9	6.3	4.8	5.5	3.7	5.0
**Pts**	**CD44 T3**	**CD44v6 T3**	**CD24 T3**	**CD9 T3**	**CD81 T3**	**EpCAM T3**	**Integrin α6 T3**	**Integrin β4 T3**	**Tspan8 T3**	**CXCR4 T3**
**1**	3.9	2.0	2.6	12.0	2.4	17.1	3.7	5.3	1.4	17.2
**2**	6.2	5.1	4.7	10.5	4.9	20.7	6.1	11.2	2.9	17.6
**3**	3.4	1.7	3.2	7.3	3.6	16.3	2.5	4.8	1.4	15.3
**4**	2.7	2.0	2.7	13.9	3.1	13.5	4.0	4.4	1.3	14.8
**5**	2.1	5.4	3.0	26.8	5.3	1.7	1.6	1.0	3.4	8.0
**6**	3.5	4.1	1.8	32.1	4.7	2.4	1.8	1.1	1.8	6.8
**7**	5.2	1.9	1.8	12.6	5.7	1.3	5.3	1.9	0.9	8.4
**8**	NA	NA	NA	NA	NA	NA	NA	NA	NA	NA
**10**	11.9	4.0	4.1	10.8	11.4	1.3	3.6	3.0	3.0	3.3
**11**	NA	NA	NA	NA	NA	NA	NA	NA	NA	NA
**12**	2.0	2.6	2.0	4.9	2.6	2.0	0.9	4.3	2.1	1.1
**13**	4.6	3.3	7.1	30.0	8.9	2.0	1.1	1.7	2.2	1.8
**14**	1.9	3.3	2.8	15.1	4.6	1.4	1.5	1.4	1.5	1.4
**15**	NA	NA	NA	NA	NA	NA	NA	NA	NA	NA
**16**	4.0	3.1	7.1	21.4	5.5	1.8	2.3	2.0	2.6	1.9
**17**	3.1	4.3	9.1	19.5	7.3	2.3	3.3	1.6	3.2	2.1
**18**	6.5	2.8	4.8	23.1	9.7	4.4	6.2	5.2	3.5	3.9
**19**	7.5	3.0	5.2	30.5	8.4	5.6	5.9	4.6	3.7	4.4

NA: Not applicable (as in missing value)

In 18/19 (94%) patients the blood sample that was collected at timepoint 0 (baseline) yielded meaningful results in terms of exosomes evaluation. Only in 15/19 (80%) patients we were able to perform the blood sample after 3 months of treatment. In one patient, due to early disease progression (and death), it was not possible to repeat blood sample collection and subsequent analysis. In 3 additional patients it was not performed blood sample collection after 3 months (due to early progression and subsequent loss at follow-up).

**Fig 1:**
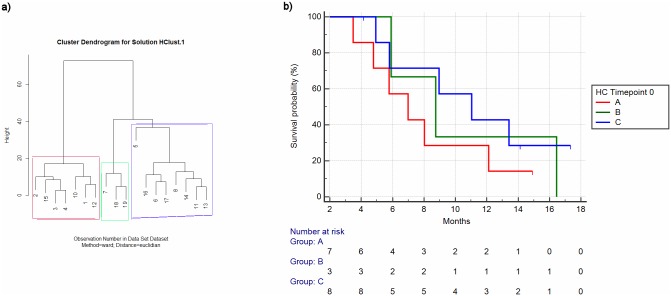
Hierarchical clustering for timepoint 0 (Numbers refer to patients as per Table 1) (a) and relative overall survival among clusters (b).

We performed hierarchical clustering analysis on sample T0 by using as stratifying factors the expression values of examined proteins (CD9, CD81, EpCAM, Integrin α6, Integrin β4, CD44, CD44v6, CXCR4, TSPAN8) and we observed three different clusters (Fig 1a).

**Fig 2:**
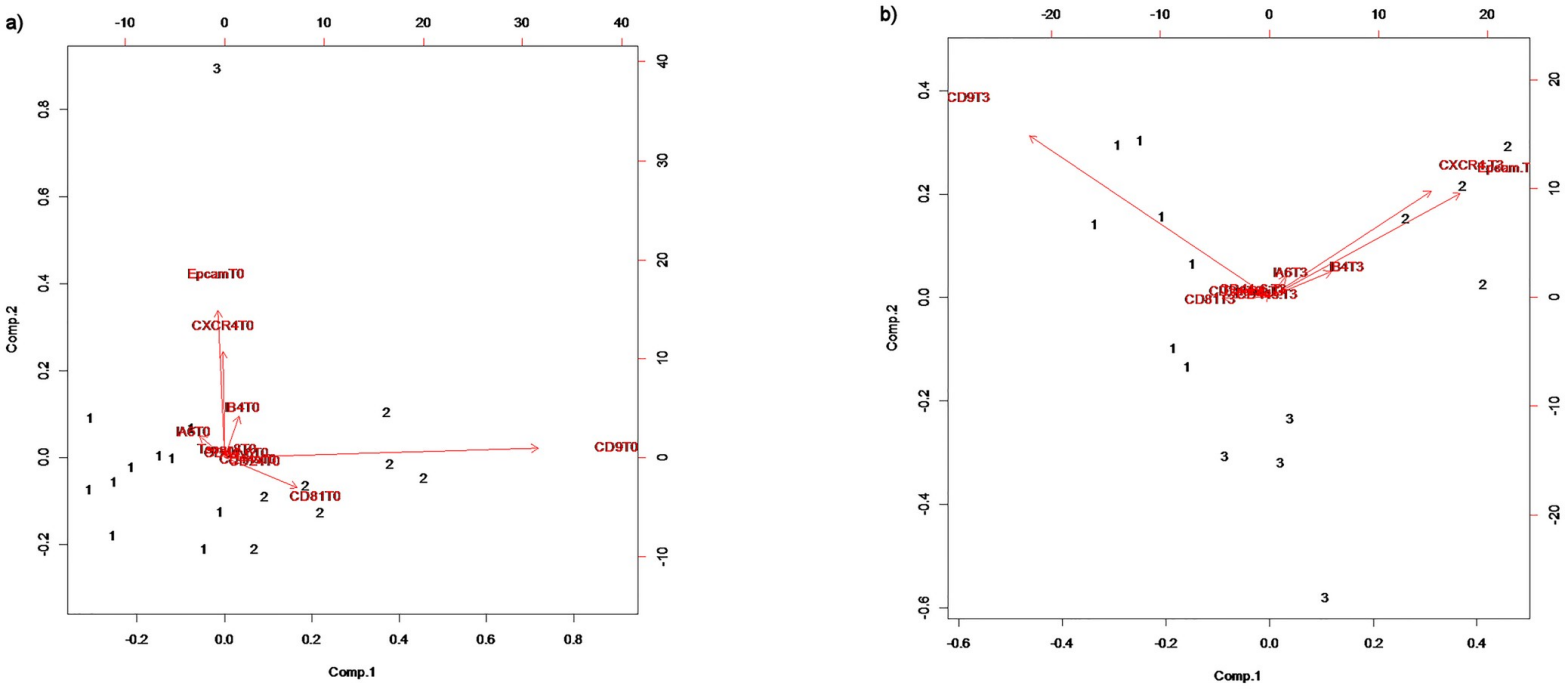
K-means (K = 3) for different exosomes’ proteins expression at timepoints 0 (a) and 3 (b).

K-means analysis (K = 3 means 3 different clusters) identified as main determinants of different allocation among the three groups the different expression of CD9 and CD81 for one group whereas the other group allocation was influenced by EpCAM and CXCR4 expression (with Integrin α6 and β4 having a minor role). Different expression of CD44, CD24, CD44v6, TSPAN8 did not influence allocation among three different groups. (Fig 2a).

**Fig 3:**
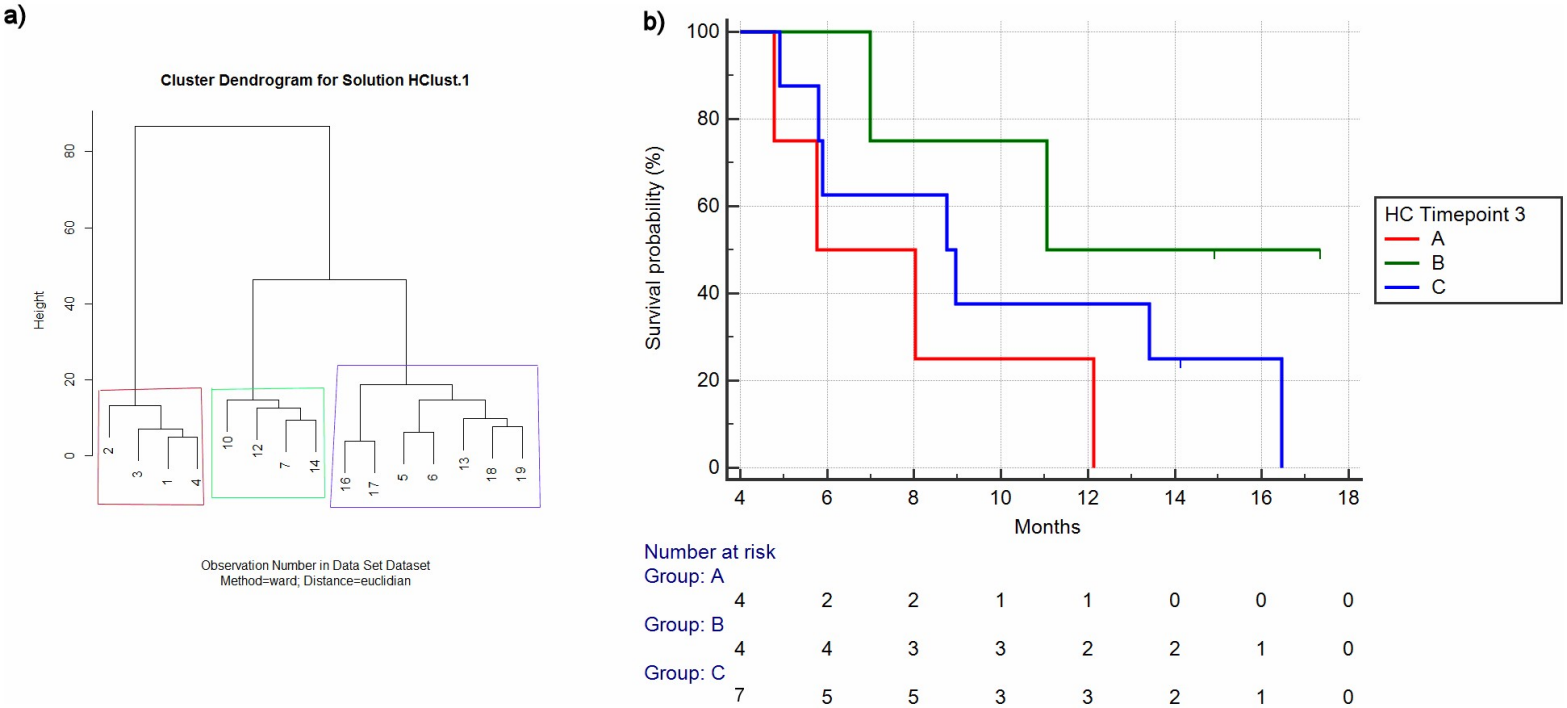
Hierarchical Clustering for timepoint 3 (Numbers refer to patients as per Table 1) (a) and relative overall survival analysis for different clusters (b).

The same results were obtained when performing hierarchical clustering analysis at T3 (Fig 3a).

K-means analysis (K = 3) revealed that CD9 and CD81 were associated with one group, expression of EpCAM, CXCR4 with another group and Integrin α6 and Integrin β4 were marginally associated (Fig 2b).

Then we performed three different analyses as to identify the correlation between exosome expression and different patient outcomes:

The first analysis was conducted by stratifying patients on the basis of CD81, CD9, EpCAM and CXCR4, as they were proven to be associated with different clusters by K-means analyses. Median concentration of the selected marker was used as cut-off point.

We analysed the correlation between 1^st^ line progression free survival (PFS) and EpCAM exosome levels at T0 and we observed a statistically significant correlation with worse PFS. In particular, patients having high EpCAM exosome expression had median PFS of 3.18 vs 7.31 months of patients having lower EpCAM levels (HR:2.82, 95%CI:1.03–7.73, p = 0.01).

**Fig 4:**
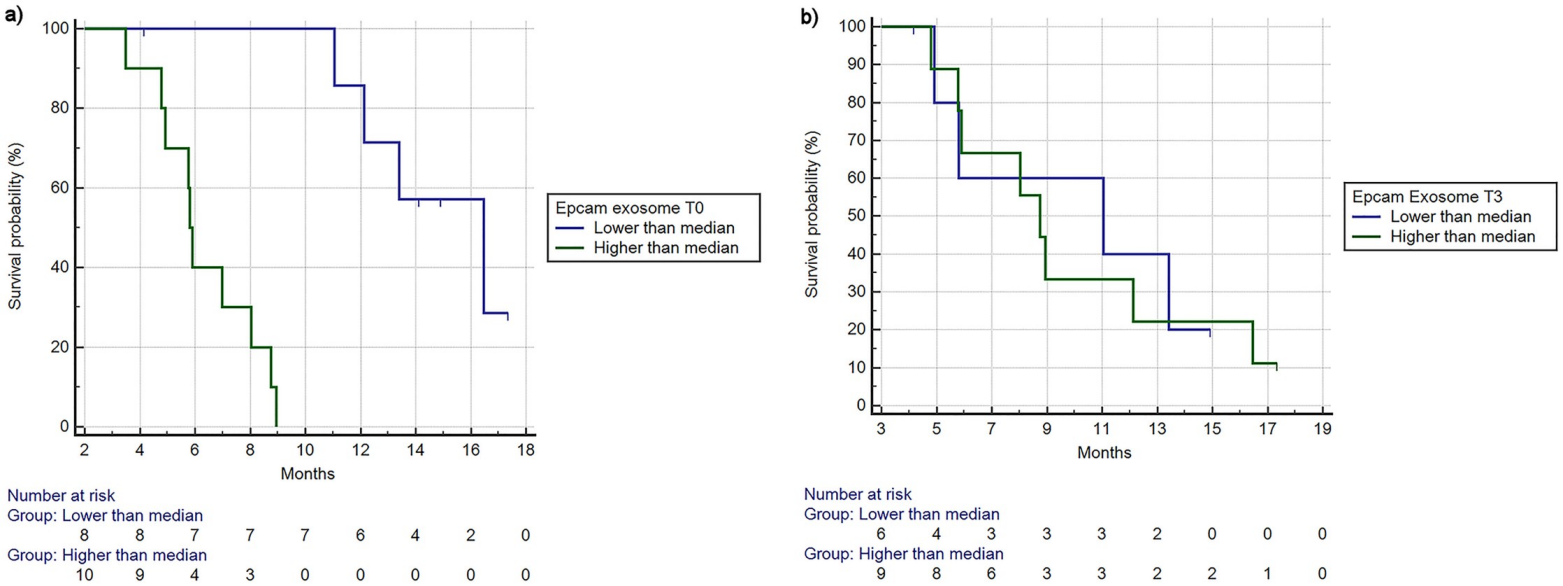
a. Overall survival stratified by high/low levels of Epcam exosome expression (timepoint 0). mOS respectively 5.83 vs 16.45 months, HR:6.16, 95%CI:1.93–19.58, p = 0.0001). b. Overall survival stratified by high/low levels of Epcam exosome expression (timepoint 3). mOS for high vs low respectively 8.75 vs 11.04 months, HR:1.10, 95%CI:0.34–3.60, p = 0.86.

In the same way, overall survival was also significantly shorter for patients having higher Epcam exosome levels(5.83 vs 16.45 months, HR:6.16, 95%CI:1.93–19.58, p = 0.0001) (Fig 4a).

There was also a statistically significant association with increased EpCAM exosome expression and lower response rate, with patients having high levels of EpCAM having 20% response rate vs 87% response rate of the low EpCAM levels group (p = 0.015).

Same trend, but not statistically significant, was observed when the same analyses were performed at T3. In particular, worse overall survival (mOS respectively 8.75 vs 11.04 months, HR: 1.10, 95%CI:0.34–3.60, p = 0.86) (Fig 4b) and shorter PFS (mPFS respectively 3.80 vs 5.73 months, HR:1.38, 95%CI:’0.43–4.39, p = 0.53) were seen for patients having higher EpCAM T3 levels. Response rates were respectively 33% vs 67% for high vs low EpCAM T3 levels. This difference was not statistically significant (p = 0.31).

CXCR4 expression at T0 was not associated with differences in terms of overall survival (higher than median cut-off vs low, mOS respectively 8.95 vs 6.98, HR:1.02, 95%CI:0.35–2.95, p = 0.96), progression free survival (mPFS respectively 6.29 vs 3.21, HR:0.42, 95%CI:0.14–1.23, p = 0.05) and response rates were 50% vs 50% for high vs low (p = 1). CXCR4 expression at T3 was also not associated with differences in terms of overall survival (high vs low, mOS respectively 8.03 vs 8.95, HR:1.93, 95%CI:0.61–6.08, p = 0.24), progression free survival (6.29 vs 3.23 months, HR:0.70, 95%CI:024–2.04, p = 0.49) and response rates (50% vs 50%, p = 1).

CD9 expression at T0 was also not associated with differences in terms of overall survival (high vs low, mOS respectively 8.75 vs 8.03 months, HR:1.31, 95%CI:0.45–3.79, p = 0.62), progression free survival (3.21 vs 6.16 months, HR:1.31, 95%CI:0.50–3.40, p = 0.56) and response rates (50% vs 50%, p = 1).

Finally, CD81 expression at T0 was not associated with differences in terms of overall survival (high vs low, mOS respectively 8.95 vs 8.03 months, HR:0.81, 95%CI:0.28–2.34, p = 0.70), progression free survival (5.78 vs 3.80 months, HR:1.18, 95%CI:0.46–3.07, p = 0.70) and response rates (66% vs 33% for high vs low respectively, p = 0.34).

The second analysis aimed at assessing whether patients identified as belonging to different clusters as indicated by hierarchical clustering at T0 and T3 showed different survival outcomes.

As per patients identified by hierarchical clustering analysis at T0, a trend towards different overall survival was seen (mOS respectively 6.98 vs 8.75 vs 11.04 months) albeit it was not statistically significant (p = 0.46) (Fig 1b). When the analysis was conducted on patients stratified by the results of hierarchical clustering analysis at T3, a more evident difference among the overall survival curves was seen (mOS respectively 5.77 vs 11.04 vs 8.75 months) but still, the difference was not statistically significant (p = 0.14) (Fig 3b).

The third analysis was conducted to identify whether patient outcomes can be predicted on the basis of increased or decreased levels of exosomal proteins between T0 and T3. Percentages of the changes in the time lapse from T0 and T3 were calculated.

**Fig 5:**
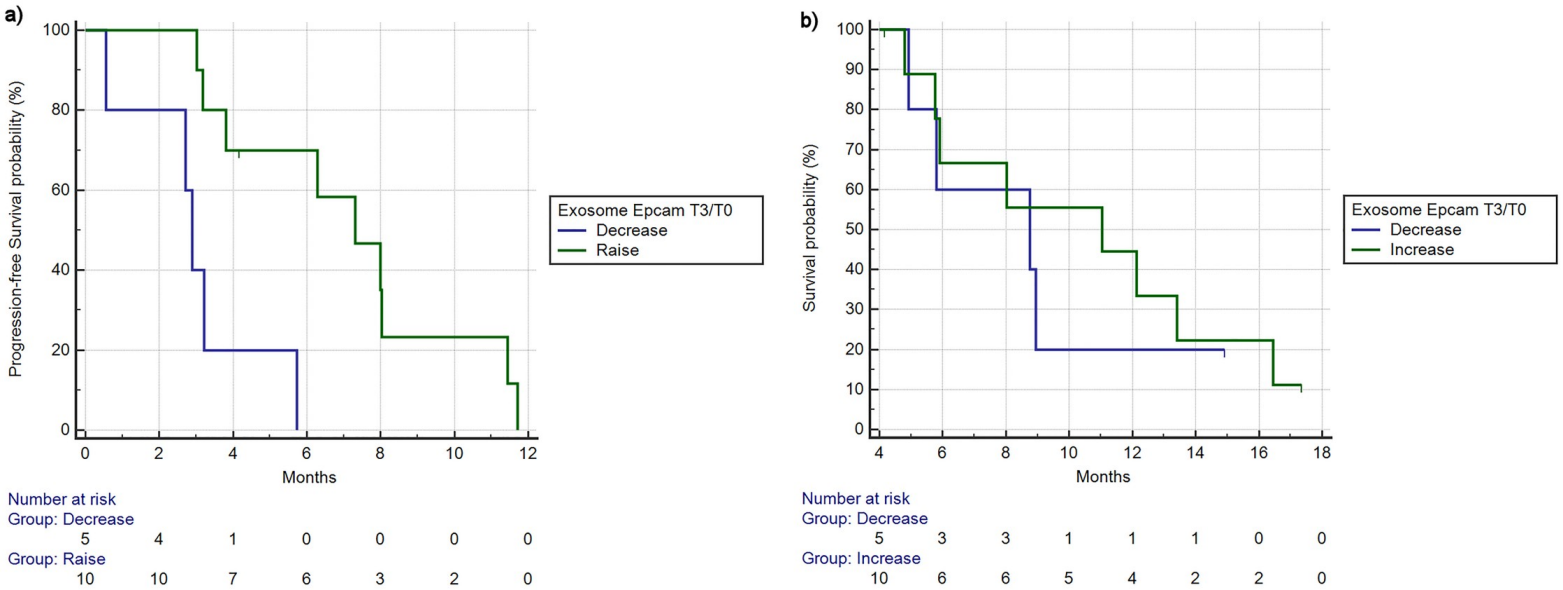
Progression free survival (a) and overall survival (b) based on changes from timepoint 3 and 0 of exosome Epcam levels (reduction/increase as % from timepoint 0 to timepoint 3). a) mPFS 2.88 vs 7.31 months, HR:0.24, 95%CI:0.04–1.22, p = 0.003). b) mOS 8.75 vs 11.04 months, HR:0.77, 95%CI:0.21–2.73, p = 0.66).

EpCAM increased levels during treatments resulted to be significantly associated with better PFS (mPFS 2.88 vs 7.31 months, HR:0.24, 95%CI:0.04–1.22, p = 0.003) (Fig 5a) and with better, but not statistically significant, OS (8.75 vs 11.04 months, HR:0.77, 95%CI:0.21–2.73, p = 0.66) (Fig 5b). Finally, response rates in the group of patients who experienced an increase in EpCAM levels were 60% vs 20% of the group of patients with decrease of EpCAM levels, albeit this difference was not statistically significant (p = 0.28).

### 3.3 Associations between exosome analyses and clinical factors

The ECOG scale of Performance Status (PS) is the strongest clinical factor as it can influence patient outcomes at the end of the therapy and overall patients’ compliance to treatment. Therefore, we assessed whether correlation with worse PS (and thus greater tumour load) could be found with different levels of those exosomal proteins resulted to stratify patients in different clusters after hierarchical cluster analysis: EpCAM, CXCR4, CD81 and CD9.

EpCAM T0 levels were correlated with worse PS. In particular, in 10 asymptomatic patients (ECOG PS:0), 7/10 (70%) had EpCAM T0 values lower than median compared with 3/10 (30%) who had high EpCAM T0 values. In 8 symptomatic (ECOG PS:1–2) patients, EpCAM T0 levels were higher than median in 7/8 (87%) vs 1/8 (13%) where low EpCAM levels were seen. This difference was statistically significant (p = 0.02 at Fisher exact test).

CXCR4 T0 levels were not correlated with different PS. In 10 ECOG PS:0 patients, 5 (50%) had high levels of CXCR4 while the remaining 5 (50%) had low levels of CXCR4. Conversely, in 8 ECOG PS:1–2 patients, 5/8 (62%) had high CXCR4 levels whereas the remaining 3/8 (38%) had low levels of CXCR4. This difference was not statistically significant (p = 0.66).

CD81 T0 levels were not correlated with different PS. In 10 ECOG PS:0 patients, 6 (60%) had low levels of CD81 whereas the remaining 4 (40%) had high levels of CD81. On the other hand, in 8 ECOG PS:1–2 patients, 3/8 (37%) had low CD81 levels whereas 5/8 (63%) had high CD81 levels. This difference was not statistically significant (p = 0.63).

CD9 T0 levels were not correlated with different PS. In 10 asymptomatic patients, 5 (50%) had low and 5 (50%) had high levels of CD9. In 8 ECOG PS:1–2 patients, 5/8 (62%) had high levels of CD9 vs 3/8 (38%) where CD9 levels were lower than median. This difference was not statistically significant (p = 0.66).

We did not find any association between different baseline CA19.9 and carcinoembryonic antigen (CEA) levels and different levels of expression of EpCAM, CXCR4, CD81 and CD9. The majority of patients was diagnosed with liver metastases but we did not find any statistically significant association between EpCAM, CXCR4, CD81 and CD9 levels and the presence/absence of liver metastases.

We did not demonstrate a statistically significant correlation between EpCAM changes during first line therapy and outcome in 2^nd^ line therapy. However, there was a trend towards worse 2^nd^ line PFS in patients who had an increase in EpCAM levels during 1^st^ line therapy (mPFS respectively 1.57 vs 2.62 months, HR:2.01, 95%CI:0.53–7.62, p = 0.30) and there was also a trend towards worse 2^nd^ line OS in patients who had an increase in EpCAM levels during 1^st^ line therapy (mOS respectively 2.23 vs 5.24 months, HR:1.85, 95%CI:0.49–6.94, p = 0.36).

Finally, when we performed multivariate analysis by using in the model all those variables who resulted to be significantly related to differences in PFS (ECOG PS, baseline EpCAM, changes in EpCAM levels from T0-T3), all variables maintained their statistical significance (respectively 0.0261, 0.0027, 0.0259). The factor that resulted to be mostly associated with differences in terms of outcome was the change in EpCAM levels between T0-T3 (Exp(B):14.28 for decrease vs increase), followed by high baseline levels of EpCAM (Exp(B):5.74) and then by ECOG PS (Exp(B):5.28).

## 4. Discussion

Patients with metastatic or locally advanced PDAC exhibit high heterogeneity in clinical behaviour, therefore the identification of prognostic biomarkers might be helpful for the choice of palliative treatments.

ASCO, ESMO and AIOM guidelines suggest that patient’s PS and age might help clinicians in choosing which kind of palliative treatment should be offered to these patients among Gemcitabine monochemotherapy, combination therapy with Gemcitabine+Nab-Paclitaxel, FOLFIRINOX or best supportive care.

However, ECOG PS is a relatively subjective form of assesment and it might deteriorate rather quickly during the course of the disease, thus reducing its reproducibility. Furthermore, age is not such a strong indicator of poor survival as it is correlated with increased toxicity (rather than lack of effectiveness) from different types of treatments.

Radiological assesment in pancreatic cancer is rarely fully informative, due to the frequent over or underestimation of the real size of the primary tumour due to the intrinsic limit of standard CT scan in defining differences in density between tumour tissue and the surrounding pancreatic tissue (that is quite frequently involved by a fibrotic process due to concomitant history of chronic pancreatitis) [14, 15].

Widely used tumour serum markers, such as CA19-9 or CEA, lack sensitivity and specificity since their levels are often normal in PDAC early stages or are falsely high in individuals with other pathological conditions (due to concomitant obstruction of the intrahepatic bile ducts caused by tumours located in the pancreatic head, usually associated with jaundice and high bilirubin levels) [16].

For these reasons the research of alternative biomarkers that could be non-invasively assessed is gaining great interest. Body fluids are enriched in exosomes, which carry on their membrane specific proteins that allow distinguishing those exosomes derived from different populations of cancer cells.

In our work, we analysed plasma samples from PDAC patients, with the aim of finding a correlation between different kinds of exosomes and patient clinical data that might be informative as prognostic and diagnostic tools. We evaluated, through our ELISA assay, the relative quantities of exosome tumor markers in enrolled patients before and after chemotherapy treatments.

Our results suggest that quantification of EpCAM levels from blood-derived exosomes might have a reliable role as prognostic factor. Highest EpCAM T0 levels were seen in patients with poor performance status and they were associated with worse PFS and OS. Conversely, in patients where we observed an increase in EpCAM levels during treatment it was observed better PFS and OS. All these factors maintaned their impact on prognosis after multivariate analysis and in particular, the strongest factor that was associated with worse/better prognosis was the change in EpCAM levels during treatment.

EpCAM (Epithelial cell adesion molecule) is a glycoprotein involved in cell-signaling, migration, proliferation and homotypic cell-cell adhesion in epithelia. It has been found almost exclusively in epithelia and in epithelial-derived neoplasms and thus it has been suggested as potential diagnostic marker for breast cancer, colorectal cancer and squamous cervical cancer[17–19]. EpCAM is a known marker of cancer stem cells in pancreatic, liver, colorectal and breast cancers [20]. EpCAM, together with CD44v6, has been shown to have an increased expression in Panc1 cancer stem-like cells. Recently, a proteomic analysis of exosomal membrane has revealed that EpCAM is one of the PDAC biomarker candidates, together with CD151, in liquid biopsies from patients [21]. Interestingly, EpCAM, together with Tspan8 and CD44v6, resulted to be one of the surface proteins found to be specifically expressed in exosomes isolated from serum samples of PDAC patients and not from healthy donors [22]. It has also been suggested that this molecule might also have a role in some cancer types (mainly those arising from the GI tract such as pancreatic, colon, gastric cancer) by silencing MSH2 gene due to processes of hypermethylation and thus causing a peculiar phenotype that is associated with altered microsatellite instability [23]. This finding might be of potential therapeutic indication as it would enable drugs such as Nivolumab or Pembrolizumab (that have been demonstrated to be active in tumours who are MSI-H) to work also in tumours that are EpCAM “impaired” or defective.

Contrary to our results, previous study has detected circulating EpCAM in all serum fraction collected from PDAC patients but it did not result a sensitive tool able to distinguish patients from healthy donors [24]. Moreover, increased levels of EpCAM mRNA extracted from circulating tumor cells, in PDAC patients after pancreatectomy, did not correlated with worse prognosis [25]. It should be noted that these results are not referred to the exosomal fraction of blood samples and this could explain the inconsistency with our results.

Our findings would suggest that EpCAM expression in circulating exosomes can have a potentially useful prognostic role. Looking at the results of the analysis it would seem that, albeit high levels of EpCAM at baseline should be correlated with worse prognosis, in those patients where actually an increase in EpCAM is found (starting from baseline up to 3^rd^ month of treatment), there is actually a better prognosis.

Although the negative prognostic role of high EpCAM baseline levels can partly be explained by greater tumour burden and corresponding worse performance status at diagnosis of the patients that were enrolled, it remains to be explained why, particularly in those patients who experienced partial response or disease stabilisation (and thus who benefited the most from palliative chemotherapy and that would have had less tumour burden after treatment), the INCREASE in exosomal EpCAM is associated with better survival outcomes. This is even more crucial if we consider that at multivariate analysis,it resulted to be the factor that was more strongly associated with better survival outcomes.

There might be 2 potential explanations of this findings, one from a statistical point of view and another from a biological point of view:

As for the statistical explanation of the finding, it can be expected that better survival outcomes in patients who had changes between 3^rd^ month of treatment and baseline could be simply related to the fact that all those patients who had a baseline blood sample and were not able to be sampled again at 3 months of treatment (due to disease progression earlier than 3 months or death) exited from the analysis BEFORE the 3^rd^ month timepoint and thus, the population of patients who are able to perform both baseline and 3^rd^ month sampling has in itself a better prognosis compared with the population of patients who have performed baseline sample (as in the fact that the second population also includes patients who are lost at follow-up or died). This might be particularly true for patients who have metastatic pancreatic cancer and thus might NOT have in a sizable proportion of cases life expectancy of 3 months. To further confirm whether this proves true it would be necessary to increase the number of patients assessed and with an earlier detection of the changes in circulating exosomal EpCAM (p.e. 1-month timepoint).

From a biological point of view it is particularly intriguing the fact that better prognosis is associated with an INCREASE in EpCAM levels. Indeed, while it is known that high levels of EpCAM in epithelial cells is usually associated with an increase in proliferative activity and decrease in differentiation it is also known that cells who undergo the process of epithelial-to-mesenchymal-transition (EMT) [26] and that are usually associated with a worse clinical behaviour and less susceptibility to chemotherapy (EMT) usually have EpCAM downregulation [27]. It would be interesting to assess if, in patients who are actually receiving palliative chemotherapy, the presence of EpCAM in circulating exosomes would detect that number of tumours where EMT is not happening and thus suggest better prognosis for these patients. To further confirm this it would be necessary to see whether, particularly in metastatic sites of involvement, EpCAM expression is different in tumours who have a decrease in exosomal EpCAM levels vs those where an increase is seen and whether this correlates with different prognosis. However, difficulties in performing repeated biopsies in metastatic sites of pancreatic cancer patients who are receiving palliative treatment and the heterogeneity of metastatic involvement, poses a major issue in the actual realisation of this kind of test.

Enzyme linked immunoassay (ELISA) is a commonly used method for protein detection and quantification. This method results in an elevated efficiency of recovered exosomes from complex matrices like body fluids. It is highly specific due to the antibodies that can identify one of the different proteins expressed on exosome surfaces. ELISA assay has been demonstrated to be a robust method for the detection and quantification of disease-derived exosomes in human biological samples and tumour models.

In this work, we have first optimized the ELISA protocol in order to isolate the exosome fraction from human plasma samples and to maximise the signal of plasma samples reducing the background noise. Unfortunately, the high sensitivity of this method is also a drawback, since even small difference of plate conditions could affect ELISA results.

This, together with the small number patients enrolled, is the principal limitation of our study.

In conclusion, this study has provided a first approach for analysing, in plasma samples, changes of exosome levels and comparing them with the disease status of patients in order to find candidate biomarkers of PDAC and potential new therapeutic targets.

On these bases we believe that exosome analysis focused on EpCAM could prove to be potentially useful both as as prognostic factor or as a target for new treatment modalities in metastatic pancreatic cancer patients since drugs such as Edrecolomab and Catumaxomab, targeting EpCAM, are currently under investigation in a series of cancer types) [28].

## Supporting information

S1 File Informed consent. (ZIP)

S2 File Patients’ database. (ZIP)

## Abbreviations

95%CI: 95% Confidence Interval
CT: Computerised Tomography
D-MMR/P-MMR: MisMatch Repair proteins expression—Defective or Proficient
ECOG PS: Eastern Cooperative Oncology Group Performance Status
EMT: Epithelial-to-mesenchymal transition
GI: GastroIntestinal
HR: Hazard Ratio
LAPC: Locally Advanced Pancreatic Cancer
MSI-H/MSI-L: MicroSatellite Instability—High or Low
OS: Overall Survival
PDAC: Pancreatic Ductal AdenoCarcinoma
PEXG: Cisplatin + Epirubicin + Capecitabine + Gemcitabine
PFS: Progression Free Survival
RECIST: Response Evaluation Criteria In Solid Tumours
RR: Response Rate
