# Supplementary material for: Clinical impact of different exosomes’ protein expression in pancreatic ductal carcinoma patients treated with standard first line palliative chemotherapy
Source: PLoS One. 2019 May 2;14(5):e0215990. doi: 10.1371/journal.pone.0215990 (PMC6497273; doi:10.1371/journal.pone.0215990)
Supplement: S1 File — (ZIP) [file pone.0215990.s001.zip › S1 FILE/Informed Consent Page 1.pdf]

Azienda Ospedaliera Universitaria Ospedali Riuniti
Clinica di Oncologia Medica
Ancona

Cellule tumorali circolanti ed esosomi nel carcinoma pancreatico. Impatto sulla prognosi e sulla strategia di trattamento

CONSENSO INFORMATO

NUMERO PROTOCOLLO:

TITOLO: Cellule tumorali circolanti ed esosomi nel carcinoma pancreatico. Impatto sulla prognosi e sulla strategia di trattamento
NOME ISTITUTO: AOU Ospedali Riuniti

INDIRIZZO: VIA CONCA 71, ANCONA

NOME DEL PAZIENTE:

Egregio Sig.re/Sig.ra
Le stiamo chiedendo la disponibilità a partecipare ad uno studio osservazionale rivolto ai pazienti affetti da neoplasia pancreatica.
La Sua partecipazione a questo studio è del tutto volontaria. La Sua decisione di prendervi parte o meno non avrà nessuna conseguenza su di Lei, né sulle cure mediche che riceve. Se decidesse di partecipare, sarà libera/o di ritirare il Suo consenso e di interrompere la Sua partecipazione in qualsiasi momento, senza che ciò abbia alcuna conseguenza su di Lei o sulla qualità delle cure
