# Supplementary material for: Clinical impact of different exosomes’ protein expression in pancreatic ductal carcinoma patients treated with standard first line palliative chemotherapy
Source: PLoS One. 2019 May 2;14(5):e0215990. doi: 10.1371/journal.pone.0215990 (PMC6497273; doi:10.1371/journal.pone.0215990)
Supplement: S1 File — (ZIP) [file pone.0215990.s001.zip › S1 FILE/Informed Consent Page 2.pdf]

mediche che riceverà. Se decidesse di partecipare a questo studio, dovrà firmare il presente modulo per dimostrare il suo consenso[1].

Introduzione

Lei è stato scelto/a per questo studio perchè affetto da carcinoma pancreatico. Le cellule tumorali circolanti e gli esosomi, piccole vescicole contenenti RNA, sono state proposte come possibili meccanismi coinvolti nei processi di metastatizzazione del carcinoma pancreatico.

Lo scopo dello studio al quale la invitiamo a partecipare è valutare se le cellule tumorali circolanti e gli esosomi siano correlati al rischio di ricaduta di malattia in seguito ad intervento chirurgico per carcinoma pancreatico, oppure al rischio di progressione nei pazienti in trattamento per malattia avanzata e dimostrare pertanto se possano rappresentano dei biomarcatori in grado di guidare la strategia di trattamento.

Quello che Le chiediamo è pertanto di sottoporsi a dei prelievi ematici specifici, assieme o aggiuntivi oltre a quelli già previsti per il monitoraggio dei parametri biochimici in corso di follow up per carcinoma pancreatico. Lo studio prevede inoltre un'analisi molecolare del tessuto tumorale asportato durante l'intervento chirurgico per valutare l'espressione di alcuni geni potenzialmente coinvolti nei processi di metastatizzazione. Le chiediamo pertanto l'autorizzazione ad utilizzare una parte del tessuto tumorale asportato durante l'intervento chirurgico, quindi senza sottoporsi ad biopsie aggiuntive, per tale analisi.

Calendario dei prelievi

Se deciderà di partecipare a questo studio, Lei sarà sottoposta/o ai seguenti prelievi ematici:

Pazienti operati:

Prelievo 1: 7-10 giorni prima dell'intervento chirurgico
