# Supplementary material for: Clinical impact of different exosomes’ protein expression in pancreatic ductal carcinoma patients treated with standard first line palliative chemotherapy
Source: PLoS One. 2019 May 2;14(5):e0215990. doi: 10.1371/journal.pone.0215990 (PMC6497273; doi:10.1371/journal.pone.0215990)
Supplement: S1 File — (ZIP) [file pone.0215990.s001.zip › S1 FILE/Informed Consent Page 3.pdf]

Azienda Ospedaliera Universitaria Ospedali Riuniti
Clinica di Oncologia Medica
Ancona

Prelievo 2: 1 mese dopo la chirurgia

Prelievo 3: 6 mesi dopo la chirurgia

Prelievo 4: il giorno in cui si evidenzia la progressione della malattia

Pazienti non operati:

Prelievo 1: giorno di inizio della chemioterapia

Prelievo 2: 3 mesi dopo l'inizio della chemioterapia

Prelievo 3: il giorno in cui si evidenzia la progressione della malattia

I rischi connessi alla procedura dei prelievi sono dolore alla puntura della vena, solo occasionalmente ematoma locale, sempre reversibile. I risultati dei prelievi potranno rendersi disponibili anche molti mesi o anni dopo il prelievo stesso, il suo Medico potrà eventualmente comunicarle l'esito ma non è prevista al momento nessuna influenza specifica sul suo trattamento oncologico.
