# Supplementary material for: Clinical impact of different exosomes’ protein expression in pancreatic ductal carcinoma patients treated with standard first line palliative chemotherapy
Source: PLoS One. 2019 May 2;14(5):e0215990. doi: 10.1371/journal.pone.0215990 (PMC6497273; doi:10.1371/journal.pone.0215990)
Supplement: S1 File — (ZIP) [file pone.0215990.s001.zip › S1 FILE/Informed Consent Page 4.pdf]

Azienda Ospedaliera Universitaria Ospedali Riuniti
Clinica di Oncologia Medica
Ancona

Il/La sottoscritto/a: ______________________ nato/a a:______________________

in data ________________________

Dichiaro che il Dr______________________________mi ha spiegato le finalità e le procedure dello studio clinico a cui mi è stato chiesto di prendere parte.

Inoltre Dichiaro che

☐ Ho letto e compreso il foglio di informazioni che mi è stato consegnato e ho avuto tempo adeguato per decidere.

☐ Sono stato/a informato/a riguardo al fatto che la partecipazione allo studio non influenzerà le decisioni terapeutiche del Medico in merito alla mia patologia.

☐ Ho avuto l' opportunità di porre delle domande e di riflettere sulle risposte date.

☐ Comprendo che la partecipazione allo studio è volontaria e che mi posso ritirare dallo studio stesso in qualsiasi momento lo voglia.

☐ Comprendo che il presente consenso ha validità anche ai sensi e per gli effetti del D.Lgs 196/03 "Codice in materia di tutela dei dati personali" ed acconsento quindi alla verifica ed inserimento dei miei dati personali nelle banche dati concernenti lo studio predetto da parte di personale dedicato che tratterà i dati suddetti in modo strettamente confidenziale e senza alcun riferimento personale e violazione della riservatezza. Se i risultati dello studio saranno pubblicati, la mia identità non sarà mai rivelata

☐ Prendo atto che i dati oggetto della presente ricerca potranno essere trattati e/o comunicati ai delegati del Titolare del Trattamento, alle Autorità Ministeriali ed ai Comitati Etici sempre con i criteri di riservatezza e di sicurezza descritti nell'informativa e solamente per le finalità per le quali il presente consenso è stato rilasciato

☐ Sono stato informato che potrò rivolgermi in qualunque momento al Responsabile del Trattamento dei Dati per esercitare i diritti di cui all'Art.7 del D.Lgs.196/03; - Il Titolare del trattamento dei Suoi dati personali è l'Azienda Sanitaria presso la quale si svolge la
