# Supplementary material for: Clinical impact of different exosomes’ protein expression in pancreatic ductal carcinoma patients treated with standard first line palliative chemotherapy
Source: PLoS One. 2019 May 2;14(5):e0215990. doi: 10.1371/journal.pone.0215990 (PMC6497273; doi:10.1371/journal.pone.0215990)
Supplement: S1 File — (ZIP) [file pone.0215990.s001.zip › S1 FILE/Informed Consent Page 5.pdf]

Azienda Ospedaliera Universitaria Ospedali Riuniti
Clinica di Oncologia Medica
Ancona

sperimentazione, legalmente[2] rappresentata dal Direttore Generale. Il Responsabile dello Studio è lo Sperimentatore principale, nella persona del [3]Dott. Riccardo Giampieri

☐ Pertanto dò liberamente il mio consenso a prendere parte allo studio clinico. La firma su questo modulo non verrà ad incidere sui miei diritti legali.

☐ Confermo che mi è stata consegnata una copia del Foglio informativo/Consenso informato datato e firmato.

☐ In caso di ulteriori domande farò riferimento al Dr.: ______________________________

4

Firma del Pazient[illegible]_____________ Data: ________________

(o dell'eventuale rappresentante legalmente riconosciuto) *(apposta dal Paziente)*

Confermo di aver spiegato la natura di questo studio clinico al/alla paziente di cui sopra e che egli/ella ha capito le spiegazioni.

Firma del Medico:__________________________________ Data: ________________

(apposta dal Paziente)

____
